# Korsakoff Syndrome in Non-alcoholic Psychiatric Patients. Variable Cognitive Presentation and Impaired Frontotemporal Connectivity

**DOI:** 10.3389/fpsyt.2018.00204

**Published:** 2018-05-31

**Authors:** Georgios Nikolakaros, Timo Kurki, Janina Paju, Sokratis G. Papageorgiou, Risto Vataja, Tuula Ilonen

**Affiliations:** ^1^“Specialists in Psychiatry” Medical Center, Turku, Finland; ^2^Satakunta Hospital District, Psychiatric Care Division, General Psychiatry Outpatient Clinic, Pori, Finland; ^3^Turku University Central Hospital, Salo Psychiatry Outpatient Clinic, Salo, Finland; ^4^Terveystalo Pulssi Medical Center, Turku, Finland; ^5^Department of Radiology, University of Turku, Turku, Finland; ^6^Department of Psychiatry, University of Turku, Turku, Finland; ^7^Cognitive Disorders/Dementia Unit, 2nd University Department of Neurology, Medical School, National and Kapodistrian University of Athens, University General Hospital “ATTIKON”, Athens, Greece; ^8^Division of Neuropsychiatry, Helsinki University Hospital, Helsinki, Finland

**Keywords:** connectome, diffusion tensor imaging, Korsakoff syndrome, magnetic resonance imaging, memory, muscle weakness, neuropsychological tests, Wernicke's encephalopathy

## Abstract

**Background:** Non-alcoholic Wernicke's encephalopathy and Korsakoff syndrome are greatly underdiagnosed. There are very few reported cases of neuropsychologically documented non-alcoholic Korsakoff syndrome, and diffusion tensor imaging (DTI) data are scarce.

**Methods:** We report clinical characteristics and neuropsychological as well as radiological findings from three psychiatric patients (one woman and two men) with a history of probable undiagnosed non-alcoholic Wernicke's encephalopathy and subsequent chronic memory problems.

**Results:** All patients had abnormal neuropsychological test results, predominantly in memory. Thus, the neuropsychological findings were compatible with Korsakoff syndrome. However, the neuropsychological findings were not uniform. The impairment of delayed verbal memory of the first patient was evident only when the results of the memory tests were compared to her general cognitive level. In addition, the logical memory test and the verbal working memory test were abnormal, but the word list memory test was normal. The second patient had impaired attention and psychomotor speed in addition to impaired memory. In the third patient, the word list memory test was abnormal, but the logical memory test was normal. All patients had intrusions in the neuropsychological examination. Executive functions were preserved, except for planning and foresight, which were impaired in two patients. Conventional MRI examination was normal. DTI showed reduced fractional anisotropy values in the uncinate fasciculus in two patients, and in the corpus callosum and in the subgenual cingulum in one patient.

**Conclusions:** Non-alcoholic Korsakoff syndrome can have diverse neuropsychological findings. This may partly explain its marked underdiagnosis. Therefore, a strong index of suspicion is needed. The presence of intrusions in the neuropsychological examination supports the diagnosis. Damage in frontotemporal white matter tracts, particularly in the uncinate fasciculus, may be a feature of non-alcoholic Korsakoff syndrome in psychiatric patients.

## Introduction

Wernicke's encephalopathy (WE) is caused by thiamine deficiency (1, 2, 3). Undiagnosed or inadequately treated WE can cause Korsakoff syndrome (KS). KS is characterized by severe chronic cognitive impairment that predominantly affects memory (4, 5). However, evidence suggests a wide range of cognitive impairment (6–10). WE and KS are referred together as the Wernicke-Korsakoff syndrome (WKS) (6, 11). Previously, the full classic triad (ocular abnormalities, ataxia or unsteadiness, and an altered mental state or mild memory deficiency) was needed to diagnose WE. However, the diagnostic assessment proposed by Caine et al. (WE can be diagnosed with any two of four signs, any element of the classic triad and nutritional deficiency) has been gaining acceptance (2, 6, 12).

Alcoholic and non-alcoholic WE and alcoholic KS have been extensively studied, but very little is known about non-alcoholic KS. Only recently it has become clear that many patients do develop KS after non-alcoholic WE (6, 13, 14). Even so, there are only 14 previously reported cases of non-alcoholic KS with the results of the neuropsychological examination presented (15, 16). Based on autopsy studies, at least 94% of non-alcoholic WE patients are not diagnosed during life (17). Still, KS after undiagnosed non-alcoholic WE has been very rarely reported during the last few decades (15, 18–20). It has been suggested, that such cases may be encountered by psychiatrists (4).

Compared with alcoholic WKS, non-alcoholic WKS may be more common in women and younger subjects, and may have a better survival rate (6). In alcoholic KS, brain MRI often shows atrophy in the mammillary bodies, the frontal lobes and the vermis (21), whereas in non-alcoholic KS atrophy changes may be uncommon (15). In both alcoholic and alcoholic KS, neuropsychological assessment usually shows impairment of anterograde memory, whereas executive functions are typically affected only in alcoholic KS (5, 15, 16).

We have recently reported on three psychiatric patients with previously undiagnosed non-alcoholic WE that had typical KS findings in neuropsychological testing (15). Two of these patients were examined with diffusion tensor imaging (DTI), and one had damage in the uncinate fasciculus, a frontotemporal tract. We report here on three non-alcoholic psychiatric patients with a similar history of previously undiagnosed WE, chronic memory impairment, and frontotemporal tract damage. However, in the present set of patients, the neuropsychological findings were more diverse.

## Materials and methods

All patients were treated by one of the authors (GN). Similarly to our previous study (15), the patients were identified by screening psychiatric patients with memory impairment for nutritional deficiency in history. We reviewed the hospital records and the reports of all previous neuropsychological examinations. All patients have memory problems that emerged around the time of WE. Table 1 shows the WE manifestations: all patients had unsteadiness and an altered mental state, two patients had ocular abnormalities, and one patient had muscle weakness. The patients were not examined by brain MRI during the WE. A diagnosis of WKS had not been previously considered, and the patients did not receive thiamine therapy at the time of WE.

**Table 1:** Causes and manifestations of non-alcoholic Wernicke's encephalopathy in three psychiatric patients with Korsakoff syndrome.

**Patient No**.	**Age^a^ (years)**	**Cause of Wernicke's encephalopathy**	**Nutritional deficiency**			**Symptoms and signs of Wernicke's encephalopathy (Classic triad)**		
			**Duration**	**Weight loss (kg)**	**Lowest BMI**	**Ocular**	**Unsteadiness/ataxia**	**Altered mental state or mild memory impairment**
1^b^	33	Acute lymphatic leukemia	5 months	7^c^	16.9^c^	Impaired vision	Unsteadiness	Confusion, aberrant behavior, elevated mood, decreased need for sleep
2	38	Hyperthyroidism	6 months	26^c^	24.5^c^	N/A	Unsteadiness	Mild confusion
3	20	Diarrhea & vomiting	2 months	6^c^	17.8^c^	Impaired vision^c^, central skotoma^c^	Unsteadiness	Depression “disappeared”, decreased need for sleep^c^

a *Age at the time of Wernicke's encephalopathy*.

b *Patient 1 had also muscle weakness*.

c *Information from hospital records. N/A, Not available*.

Patient 1 met the Diagnostic and Statistical Manual of Mental Disorders, Fifth Edition (DSM-5) diagnostic criteria for alcohol use disorder at the age of 15–21 years, more than 10 years before the WE. There was no nutritional deficiency or WE manifestations during that period, and the patient has not used alcohol since. The other patients did not have a history of current or past alcohol use disorder. None of the patients had a history of traumatic brain injury.

### Clinical description

**Patient 1:** This female patient was self-referred at the age of 34 for symptoms of depression.

*WE and memory impairment:* At the age of 33, the patient was treated with cytostatic drugs, cortisone, radiotherapy, and bone marrow transplantation for acute lymphatic leukemia. She was on total parenteral nutrition for 3 weeks. While hospitalized, the patient experienced loss of appetite, daily vomiting, diarrhea, weight loss, and symptoms of WE, including pronounced muscle weakness (Table 1). A few months later, the patient's physical condition improved. At that time, she noticed memory problems. At the age of 36, a clinical neuropsychological examination showed deficits in working memory, attention, and verbal fluency. MRIs at the age of 36 and 40 showed a small developmental venous angioma in the posterior part of the right frontal lobe and mild ectopy of the cerebellar tonsils. Brain [18F]-fluorodeoxyglucose PET examination was normal.

*Prior psychiatric treatment:* The patient received counseling for depression and problems with alcohol during her teen years.

**Figure 1:**
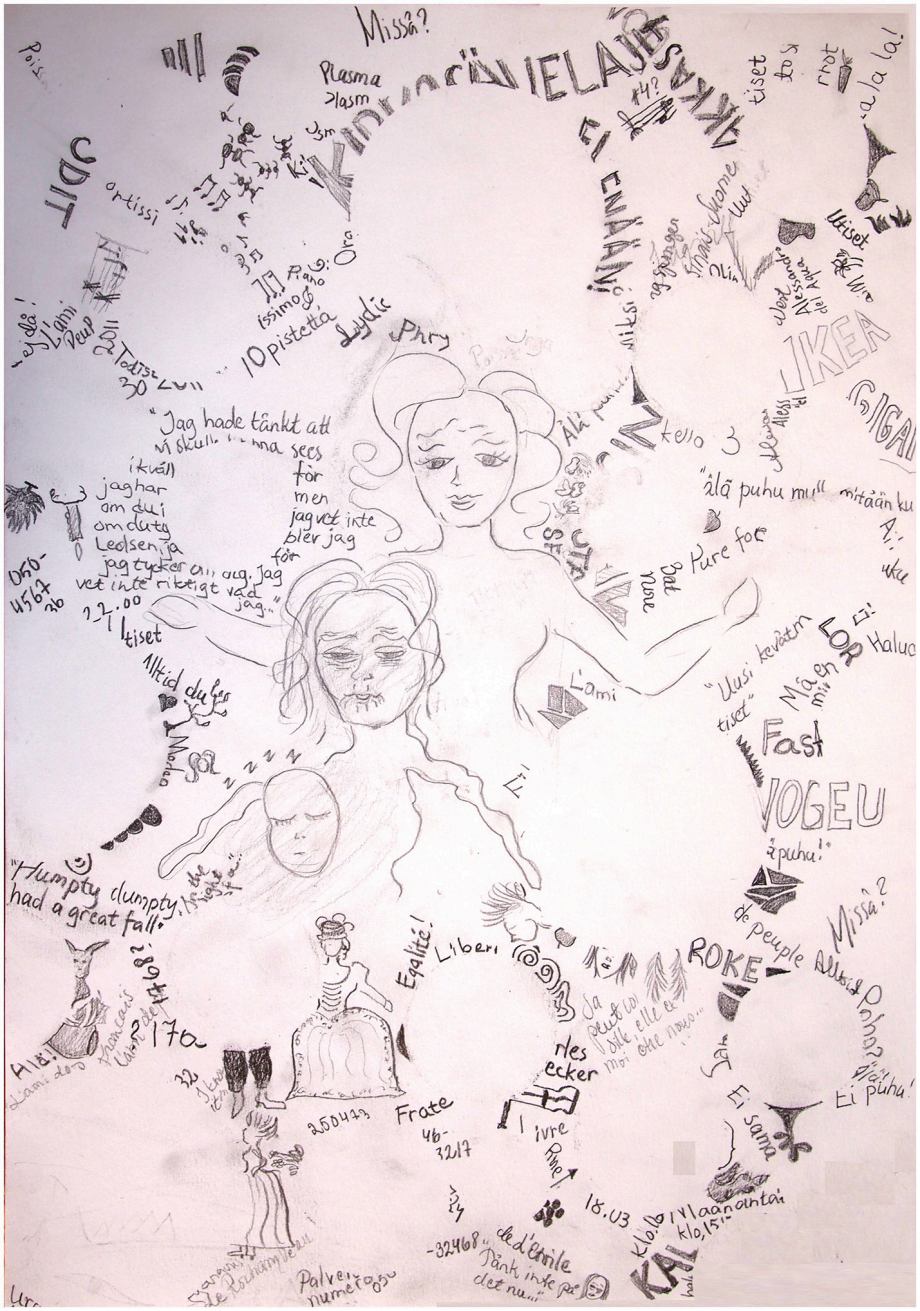
Drawing made by Patient 1 on her memory problem. The patient is writing a novel, the events take place during the French Revolution. The patient has tried to gather information on the historical background, but she keeps forgetting what she reads. The round empty spaces in the drawing represent gaps in memory. The neat and smiling female figure in the center represents the patient being diligent and in a good mood but the memory difficulties making her feel perplexed, unsure, and lost, opening her arms to apologize for the inconveniences caused. Below and to the right of this figure is an old, frail, and depressed woman, the patient when very tired and desperate by her unsuccessful attempts to learn new information, thinking of the memory problem as an old person's disease and knowing that currently there is no cure. Up and to the right of these figures, ants are taking away the patient's misplaced possessions. The musical notes represent forgetting what she had learned in music theory during a course 2 years ago. Up and on the left of the figures, the names of two large stores, the patient feels overwhelmed when she is presented with a lot of new information at the same time.

*Course and outcome:* One year after the WE, the patient was diagnosed with severe major depression. Three years later she was diagnosed with bipolar II disorder. She has also been diagnosed with generalized anxiety disorder, post-traumatic stress disorder, and obsessive-compulsive personality disorder. She had cognitive psychotherapy for 4 years and psychophysical therapy for 2 years. Current medication comprises valproate, lamotrigine, thyroid hormone for hypothyroidism, and estrogen replacement therapy. She has artisan and dance instructor's degrees and is on long-term disability leave. Currently, at the age of 43, leukemia is in remission. The patient's mood has been mostly normal, but she gets easily tired. The patient recovered from some manifestations of WE after a few weeks. However, the unsteadiness and muscle weakness have improved but are still present. The memory problem has persisted, and there is a mild confabulation tendency. Figure 1 shows a drawing made by the patient on how she experiences the memory impairment. She needs help from her parents at least four times per week to remember to eat regularly, pay her bills, find misplaced items, and cope with other daily routines.

**Patient 2:** This male software engineer was first treated by GN at the age of 51 at an outpatient psychiatric clinic.

*WE and memory impairment:* The patient received outpatient treatment with radioactive iodine for hyperthyroidism at the age of 38. His weight dropped, and he had unsteadiness (Table 1) for a few weeks. Soon after that, he started having memory problems. At the age of 41, he was diagnosed with major depression. Later, psychotic features were noted, and more recently bipolar II disorder was diagnosed. At the age of 49, a clinical neuropsychological examination showed slowness and severe memory deficit. MRI was normal at the age of 51 and 52. Brain PET examination was normal.

*Comorbidities:* The patient has used CPAP-treatment since the age of 52 for severe obstructive sleep apnea (OSA). He is also treated for hypothyroidism, high blood pressure, type 2 diabetes, and hypercholesterolemia.

*Course and outcome:* The patient has used several psychiatric medications, including quetiapine, aripiprazole, escitalopram, and bupropion. At the age of 51, he was treated with electroconvulsive therapy that did not affect the memory symptoms. He received a permanent disability pension at the age of 53. He was treated by GN for 3 years until the age of 54. At the end of the treatment, the patient's mood had partially improved, and there were no psychotic symptoms. However, the memory problem was still severe, requiring weekly home visits by a mental health nurse.

**Patient 3:** This male patient was first treated by GN at the age of 44 for major depression.

*WE and memory impairment:* At the age of 20, the patient had an antibiotic-treated infection, followed by diarrhea and vomiting for 2 weeks. He was not hospitalized at the time. The patient's appetite was very low for a further few weeks. During a period of several weeks, there were WE manifestations (Table 1), and long-term memory problems started around that time. At the age of 45, a clinical neuropsychological examination showed deficits in memory (with intrusions), attention, and executive functions; MRI and PET were normal.

*Comorbidities:* Mild OSA is treated with a mandibular advancement device.

*Prior psychiatric treatment:* The patient was diagnosed with an eating disorder during his late teens. His BMI was normal for around one year before the WE. Later, the patient was diagnosed with severe major depression, somatoform disorder, and sleep disorder.

*Course and outcome:* Since the time of WE, the patient has had daily gastrointestinal symptoms (dyspepsia, delayed emptying of the stomach). The depression has not responded to medication (paroxetine, citalopram, moclobemide, sertraline, tianeptine, agomelatine, flupentixol, modafinil) or transcranial magnetic stimulation. The memory impairment has persisted. The patient was treated by GN for 3 years until the age of 47. He has worked with media (self-learned), and he is on part-time, long-term disability leave.

### Neuropsychological assessment

Testing was performed as previously described (15). In addition, the vocabulary subtest of the Wechsler Adult Intelligence Scale-III (WAIS-III) was used to assess general cognitive level. The neuropsychological tests used are presented in Tables 2, 3. Results at least 1.5 standard deviations (SDs) below the mean of normative values were considered abnormal. We also considered abnormal a difference of 15 or more between memory quotients and the general cognitive level (22). Intrusions were defined as false words in the Hopkins Verbal Learning Test–Revised (HVLT-R) and false statements in the Wechsler Memory Scale–III (WMS-III). Intrusions semantically related to the presented material were considered semantic (23).

**Table 2:** Results of memory tests for three psychiatric patients with non-alcoholic Korsakoff syndrome.

	**Test**	**Patient 1**	**Patient 2**	**Patient 3**
Age^a^		40	53	46
**WORKING MEMORY**				
Verbal	WAIS-III^b^	−0.67 **(25)**	−1.00 (10)	1.33 (−35)
Visual	WAIS-III^c^	0.67 (5)	−1.00 (10)	1.00 (−30)
**EPISODIC MEMORY**				
**Immediate recall**				
Verbal	HVLT-R^d^	0.31 (10)	**−1.75 (21.3)**	−0.50 (−7.5)
	WMS-III^e^	**−2.00 (45)**	−1.33 **(15)**	−0.33 (−10)
Visual	BVMT-R^f^	0.10 (13.5)	−0.71 (5.7)	1.48 (−37.2)
**Delayed recall**				
Verbal	HVLT-R^g^	0.38 (9.3)	**−3.82 (52.3)**	**−1.54** (8.1)
	WMS-III^h^	−1.00 **(30)**	**−2.33 (30)**	−0.67 (−5)
Visual	BVMT-R^i^	0.31 (10.4)	−0.50 (2.6)	1.41 (−36.2)
**Verbal recognition**	HVLT-R^j^	−0.06 (14.1)	**−3.38 (45.7)**	−0.86 (−2.1)
	WMS-III^k^	**−2.00 (45)**	−1.00 (10)	1.00 (−30)

*Values outside brackets are z-scores from the distribution of normative values. Values inside brackets are the difference between the memory test quotient and the general cognitive level quotient. Values of −1.5 or less (z-score), or 15 or more (quotient difference) are in bold*.

a *Age (years) at the neuropsychological examination*.

b *Wechsler Adult Intelligence ScaleIII, letter - number span subset*.

c *Wechsler Adult Intelligence Scale–III, visual span subset*.

d *Hopkins Verbal Learning Test–Revised (HVLT-R), sum of the first three learning trials*.

e *Wechsler Memory Scale-III (WMS-III) logical memory I*.

f Brief Visuospatial Memory Test-Revised (BVMT-R), sum of the first three learning trials

g *HVLT-R, delayed free recall trial (trial 4)*.

h *WMS-III, logical memory II*.

i *BVMT-R, delayed free recall trial (trial 4)*.

j *HVLT-R, recognition trial*.

k *WMS-III, logical memory recognition score + 24 (presumed perfect Verbal Paired Associates recognition). According to Lezak, Howieson, Bigler, and Tranel, Neuropsychological assessment, 5th edition (2012), page 527*.

**Table 3:** Results of non-memory tests for three psychiatric patients with non-alcoholic Korsakoff syndrome.

	**Test**	**Patient 1**	**Patient 2**	**Patient 3**
Age^a^ (years)		40	53	46
General attention	TMT^b^-A	−0.52	**−1.64**	0.00
Divided attention	TMT^b^-B	−0.84	−0.84	3.09
Psychomotor speed	WAIS-III^c^	−0.67	**−2.00**	−0.33
Verbal skill	WAIS-III^d^	1.00	−0.33	−1.00
**EXECUTIVE FUNCTIONS**				
Planning & foresight	NAB Mazes test	**−1.64**	**−1.90**	0.20
Set shifting	WCST^e^	0.41	0.32	−1.18
Task switching	TMT(A) – TMT(B)	−0.67	−0.39	1.96
Verbal fluency	Fluency tests^f^	0.10	1.28	0.92
Composite score^g^		−0.45	−0.17	0.48

*Values are z-scores from the distribution of normative values. Values ≤ −1.5 are in bold*.

a *Age at the neuropsychological examination*.

b *Trail Making Test*.

c *Wechsler Adult Intelligence Scale–III, digit symbol subset*.

d *Wechsler Adult Intelligence Scale–III, vocabulary subtest*.

e *Wisconsin Card Sorting test*.

f *The category fluency test (animals) and the word fluency test (words starting with the letter “s”)*.

g *Average of NAB Mazes test, WCST, TMT(A)—TMT(B), and fluency tests*.

### DTI and conventional brain MRI

All patients underwent a MRI/DTI examination with similar imaging procedures and normal controls as previously described (15, 24, 25). MRI/DTI was performed at 3T (Achieva, Philips Medical Systems) using a sensitivity encoding (SENSE) 8-channel transmit-receive head coil. The imaging protocol consisted of transverse T2-weighted turbo spin echo images, coronal fluid attenuated inversion recovery (FLAIR) images, sagittal 3D FLAIR images, sagittal 3DT1 turbo field echo images, transverse susceptibility-weighted fast field echo images (venous BOLD), and transverse DTI images. Diffusion-weighted turbo spin echo EPI images (TR/TE 5877/62, 60 slices with 2.0 mm thickness, gap 0.0 mm, 112 × 128r matrix, turbo factor 59, EPI factor 59, FOV 224 mm, RFOV 100%, number of excitations 2, imaging time 3 min 52 s); b values of 0 and 800 sec/mm2 and 15 different gradient encoding directions were used, and isotropic images with 2.0 × 2.0 × 2.0 mm voxel size were obtained. The images were post processed with the Philips Diffusion Registration Tool (Philips, Medical Systems, Best, the Netherlands) to remove distortions and misalignments due to shear and eddy current, as well as head motion. All transverse images were obtained according to the line between the lower border of the genu and splenium of the corpus callosum.

Deterministic DTI tractography (FiberTrak package, Philips) was performed using the following fractional anisotropy (FA) and turning angle thresholds to terminate the tracking process: FA 0.15/angle 27, FA 0.15/angle 40, FA 0.30/angle 27, or FA 0.50/angle 27 depending on the tract. The tracts were defined by means of two or three free-hand inclusion regions-of-interest and 1-3 exclusion regions-of-interest, which were placed in standard positions by a neuroradiologist (TK) according to anatomical landmarks.

FA-values were compared to those of normal controls. The controls comprised volunteers and subjects scanned to exclude arterial aneurysms or due to benign sinonasal problems. For the subgenual cingulum and the fornix we used 16 controls for each patient, matched for sex, and in the same age range with the patients (36–55 years). For the rest of the tracts we used 30 controls matched for age and sex to each patient. The controls had normal brain MRI scans. The MRI acquisition protocol was the same for the patients and controls. A questionnaire and review of medical records were used to exclude traumatic brain injury, other neurological diseases, psychiatric disease, vascular disease, hypertension, and chronic alcoholism in the controls.

In DTI, we examined the corpus callosum (separately genu, body, and splenium), the cingulum [separately the superior and inferior (parahippocampal) sections, and in addition the subgenual part separately (26)], the uncinate fasciculus (UF), the superior and inferior longitudinal fasciculi, the left arcuate fasciculus, the inferior fronto-occipital fasciculus, the anterior corona radiata, the prefrontal projection fibers (corticostriatal projections), the projection tracts through mesencephalon, and the fornix (separately the descending fornix and the whole tract). For each tract separately, we subtracted the mean FA-value of the controls from the patient's FA-value, and we then divided this difference with the SD-value of the controls. Values ≤ −2 (representing patient FA-values at least 2 SDs below the mean of controls) were considered abnormal.

### Consent to participate

The study complied with the Declaration of Helsinki. All patients gave written consent for the use of their medical information in this publication. The Finnish legislation does not require an ethics committee's permission for studies that are restricted to the use of patients' medical information, in case the subjects have given written consent for the use of their medical information.

## Results

### Neuropsychological assessment

Table 2 shows the results of the memory tests. Delayed verbal recall was impaired in all patients, and immediate verbal recall was impaired in two patients. Two patients had impaired verbal recognition. Verbal working memory was impaired in Patient 1. Patient 1 had an abnormal result in the logical memory test (WMS-III) but not in the word list memory test (HVLT-R). Patient 3 had an abnormal result in the word list test but not in the logical memory test. The memory impairment of Patient 1 was evident only when compared with her general cognitive level. In the HVLT-R, Patient 1 had no intrusions, Patient 2 had six intrusions, and Patient 3 had one intrusion. In the WMS-III, Patient 1 had nine intrusions, Patient 2 had eight intrusions, and Patient 3 had six intrusions. All intrusions were semantic. Table 3 shows the results of the non-memory tests. Patient 1 had an abnormal result in one test of executive functions (planning and foresight). Patient 2 had abnormal results in general attention, psychomotor speed, and in planning and foresight. All other tests, including the other individual tests of executive functions and the composite score of executive functions, were normal.

### DTI (Table 4, Figure 2)

FA-values more than 2 SDs below the mean of controls were found in the corpus callosum (both genu and body), in the subgenual cingulum bilaterally, and in the UF (right side) of Patient 1, and in the UF (left side) of Patient 2. The left UF of Patient 1 and both UFs of Patient 3 had low FA-values also, but these values did not reach the −2 SD threshold. The UFs of Patients 1 and 3 and the subgenual cingula of Patient 1 were small.

**Table 4:** Fractional anisotropy (FA) values from diffusion tensor imaging for three psychiatric patients with non-alcoholic Korsakoff syndrome.

**White matter tract**	**Tractography threshold**	**Patient 1, FA-value**	**Patient 1, z-score**	**Patient 2, FA-value**	**Patient 2, z-score**	**Patient 3, FA-value**	**Patient 3, z-score**
Corpus callosum genu	0.50/27	0.583	**−2.0**	0.599	−0.7	0.598	−0.8
Corpus callosum body	0.50/27	0.589	**−2.0**	0.600	−1.3	0.603	−1.0
Corpus callosum splenium	0.50/27	0.626	−1.2	0.632	−1.0	0.636	−0.5
Superior cingulum, right	0.15/27	0.397	−1.4	0.433	0.7	0.419	−0.1
Superior cingulum, left	0.15/27	0.411	−0.9	0.428	0.1	0.437	0.4
Inferior cingulum, right	0.15/27	0.358	−1.0	0.419	1.4	0.387	−0.1
Inferior cingulum, left	0.15/27	0.371	−0.8	0.402	0.5	0.371	−0.8
Subgenual cingulum, right	0.15/27	0.309	**−2.3**	0.359	−0.3	0.372	0.2
Subgenual cingulum, left	0.15/27	0.320	**−2.1**	0.377	0.3	0.381	0.4
Uncinate fasciculus, right	0.15/27	0.349	**−2.3**	0.376	−0.7	0.361	−1.6
Uncinate fasciculus, left	0.15/27	0.375	−1.7	0.371	**−2.2**	0.377	−1.8
Superior longitudinal fasciculus, right	0.15/27	0.384	−1.0	0.387	−0.9	0.388	−0.8
Superior longitudinal fasciculus, left	0.15/27	0.400	−1.4	0.415	−0.3	0.429	0.7
Inferior longitudinal fasciculus, right	0.15/27	0.455	0.7	0.450	0.3	0.438	−0.4
Inferior longitudinal fasciculus, left	0.15/27	0.436	−0.4	0.432	−0.9	0.453	0.4
Arcuate fasciculus, left	0.15/27	0.442	−0.9	0.472	0.5	0.448	−0.8
Inferior fronto–occipital fasciculus, right	0.15/27	0.463	0.3	0.466	0.3	0.456	−0.3
Inferior fronto–occipital fasciculus, left	0.15/27	0.451	−1.0	0.469	0.1	0.479	0.7
Anterior corona radiata, right	0.15/27	0.406	−1.6	0.423	−0.5	0.440	0.6
Anterior corona radiata, left	0.15/27	0.436	−0.6	0.450	0.8	0.443	0.1
Prefrontal projection fibers^a^, right	0.15/27	0.336	−0.3	0.349	0.5	0.343	0.0
Prefrontal projection fibers^a^, left	0.15/27	0.340	−0.5	0.346	−0.1	0.353	0.4
Mesencephalic projections, right	0.30/27	0.521	−0.2	0.525	0.4	0.515	−0.7
Mesencephalic projections, left	0.30/27	0.517	−0.8	0.529	0.1	0.516	−0.8
Fornix, right	0.15/40	0.385	1.6	0.386	1.7	0.399	2.3
Fornix, left	0.15/40	0.359	−0.1	0.322	−1.7	0.361	0.1
Descending fornix, right	0.15/40	0.407	2.4	0.323	−1.4	0.363	0.4
Descending fornix, left	0.15/40	0.388	1.0	0.363	0.0	0.380	0.7

*Z-scores represent the difference from the mean of controls with one standard deviation as the unit. Values ≤ −2 are in bold*.

a *Corticostriatal projections*.

**Figure 2:**
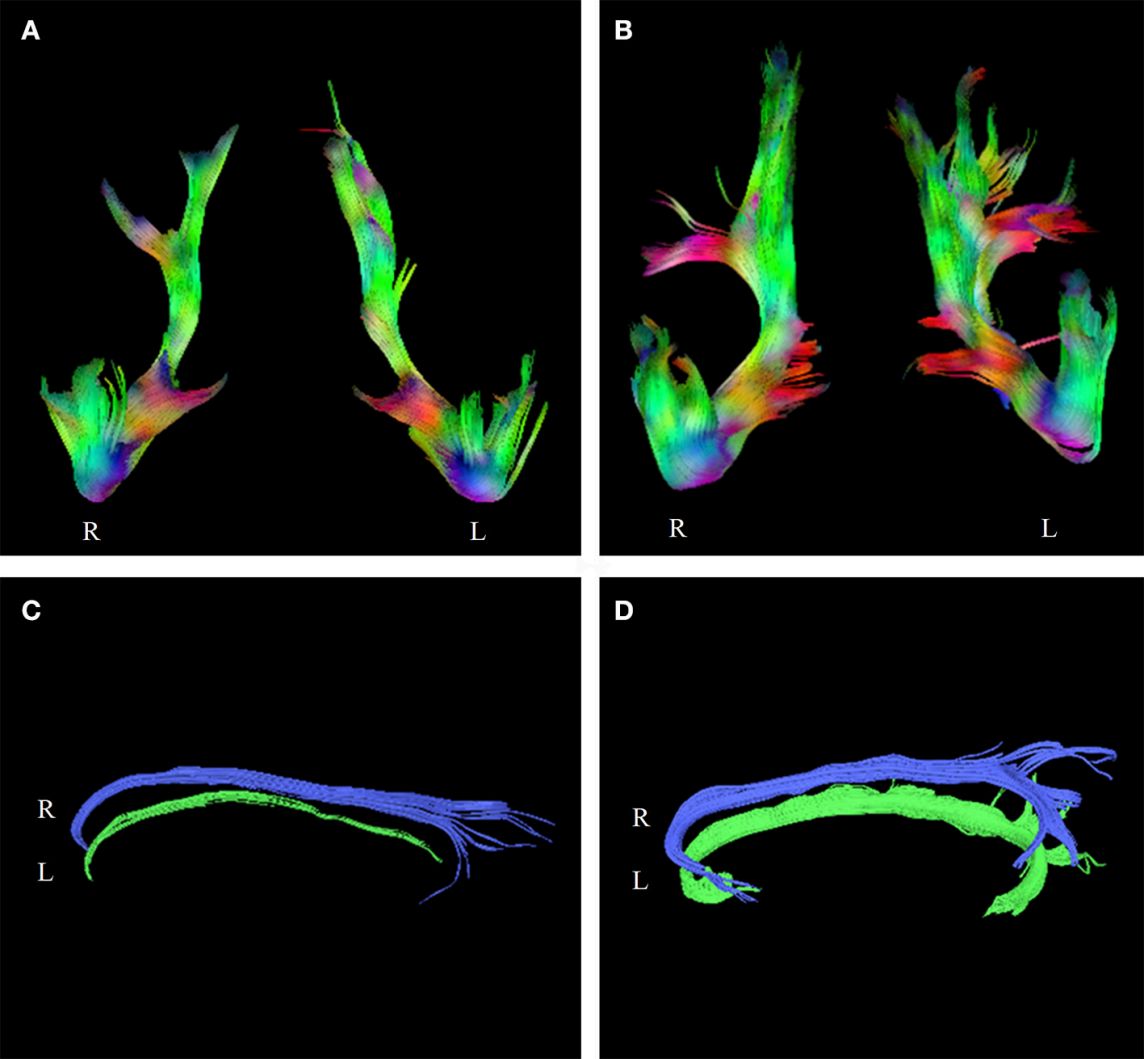
Tractograms of the uncinate fasciculi of Patient 1 **(A)** and a control subject **(B)**. Tractograms of the subgenual cingula of Patient 1 **(C)**, and a control subject **(D)**. The patients' tractograms are markedly smaller than the tractograms of the controls. R, Right side, L, Left side.

### Conventional MRI

All examinations were normal, except for the above-mentioned venous angioma and cerebellar tonsil ectopy in the MRI of Patient 1.

## Discussion

Similarly to our recent report (15), the patients in the present study had a history of probable but undiagnosed WE and subsequent long-term memory impairment with intrusions. Two patients had clear frontotemporal damage in DTI. However, the neuropsychological findings, although compatible with KS, were diverse and not entirely typical for KS. For Patient 1, the impairment of delayed memory was evident only when memory was compared to her general cognitive level. For two patients, the verbal memory impairment was present either in the word list test or in the logical memory test but not in both. For Patient 2, impairment of immediate and delayed verbal recall and recognition was pronounced, but general attention and psychomotor speed were also impaired albeit to a lesser degree. Thus, the preferential impairment of memory was not apparent.

The variable neuropsychological findings impose a critical consideration of the KS diagnosis, particularly because two of the patients have bipolar disorder, and memory impairment may be more pronounced in bipolar disorder than in major depression (27). All patients had a period of malnutrition that lasted long enough to cause thiamine deficiency (1). The malnutrition was accompanied by typical clinical manifestations of WE. Thus, the WE diagnostic criteria proposed by Caine et al. (12) are met. The history of probable WE and the subsequent long-term stable memory impairment strongly support the diagnosis of KS. Support for the KS diagnosis is also provided by the presence of many semantic intrusions in the neuropsychological examination.

The DSM-5 includes thiamine deficiency as a cause of major and mild neurocognitive disorder due to another medical condition (28). Patients 1 and 2 need assistance with everyday activities, and all patients have memory impairment that prevents them from doing regular work. According to the DSM-5, Patient 1 has moderate major neurocognitive disorder, Patient 2 has mild to moderate major neurocognitive disorder, and Patient 3 has mild neurocognitive disorder. The DSM-5 mentions KS only in the context of alcohol-induced neurocognitive disorder presenting with very severe memory deficiency and confabulations. This covers only partly the clinical spectrum of KS. It is generally accepted that non-alcoholic WE can cause KS (6, 13, 14). Many patients with KS do not overtly confabulate (4). Bowden has noted that many studies describing KS have selectively included patients with very severe memory impairment and excluded patients with clinically milder KS (7). Previous studies have often included only KS patients that were institutionalized because of their debilitating cognitive impairment (29–34), and accordingly have DSM-5 severe major neurocognitive disorder. This may help find KS patients for scientific studies, but it also might predispose researchers and clinicians to restrict their view of the spectrum of long-term WKS cognitive damage to very severe cases. The DSM-5 states that the distinction between major and mild neurocognitive disorders is inherently arbitrary, and the disorders exist along a continuum.

Executive dysfunction is common in alcoholic KS (30, 35). We have recently noted that, similarly to the present cases, most previously reported neuropsychologically documented non-alcoholic KS cases did not have severely impaired executive functions (15). Gasquoine has extended this observation by noting that in all cases with impaired executive functions, the impairment could have been caused by other factors than thiamine deficiency (16). These observations support the notion that executive dysfunction in alcoholic KS is at least partially caused by other factors than thiamine deficiency (4, 16, 31). Thus, contrary to non-alcoholic KS, in alcoholic KS everyday functioning is commonly affected not only by the memory impairment but also by executive dysfunction. Executive dysfunction can have a devastating effect on a person's everyday life, and disentangling the effects of memory and executive impairment on a patient's functioning is not possible. Therefore, non-alcoholic KS patients could be more probable to be independent in everyday life activities compared to alcoholic KS patients with similar memory impairment, and thus less likely to be diagnosed with major as opposed to minor neurocognitive disorder.

It is difficult to be sure that milder cognitive impairment after alcoholic WE is caused by the thiamine deficiency and not by chronic alcohol use. Thus, our results suggest that thiamine deficiency *per se* can cause memory impairment that is severe enough to prevent vocational independency but does not necessitate assistance with everyday activities.

Clinical, neuropsychological, and pathological studies strongly suggest that cognitive impairment in alcoholic KS can present not only as selective memory loss, by also as global impairment (7–9). In particular, the variation in the topography of anatomical lesions supports variable clinical presentation (36). Our results (Patient 2) suggest that in addition to relatively mild cognitive impairment, thiamine deficiency *per se* can also cause broad cognitive impairment. This further strengthens previous well-documented recommendations that the possibility of WKS should be thoroughly addressed when considering the possibility of alcoholic dementia diagnosis (7, 8). Patient 3 had an abnormal word list memory test result but a normal logical memory test result, and verbal recognition memory was not impaired. Similar selective impairment has been recently reported in patients with thalamic infarction sparing the mammillothalamic tract (37). Preservation of logical memory and abnormal word list test result have been associated with executive dysfunction in some (38, 39) but not all (40) previous studies. However, Patient 3 had intact executive functions, which means that the abnormal word list test result is due to primary memory impairment.

The continuity theory in its original and revised forms claims that alcohol causes a continuum of cognitive impairment (including memory), where KS is the most severe form and “uncomplicated alcoholics” are less impaired (32). Given the preferential reporting of very severe KS (7), it is conceivable that studies in support of this theory may have assumed that the diagnosis of KS requires the presence of debilitating cognitive impairment. Thus, it is possible that KS was excluded in “uncomplicated alcoholics” based on the absence of such very severe impairment. It has been recently shown that among alcoholics that are presumed not to have KS, WE manifestations are associated with more severe cognitive impairment (41). This notion, together with the fact that many patients develop alcoholic KS in the absence of a WE history (42), makes it impossible to exclude that an “uncomplicated alcoholic” has instead relatively mild KS without a prior WE diagnosis (5, 7). This in turn might suggest that to become testable, the continuity theory of alcohol-induced cognitive impairment should be further developed. Together with these recent findings, our results of milder non-alcoholic KS support the suggestion that the continuity theory could be applied to cognitive impairment caused by thiamine deficiency (5).

Our results of variable neuropsychological presentation suggest the need to comprehensively evaluate patients with suspected KS. We have recently suggested that lack of impairment of executive functions and a tendency to misattribute organic memory impairment to psychiatric disease may contribute to the underdiagnosis of non-alcoholic KS (15). Our present results extend this notion to variable neuropsychological presentation. We have recently shown that atypical presentation is associated with underdiagnosis of OSA in psychiatric patients (43).

Intrusions in memory testing [provoked confabulations (44), simple provoked confabulations (45), memory-recall-provoked confabulations (46)] may occur in normal controls (47), but they are rare (48, 49), unless after a retention interval longer than the one used in clinical assessment (44). Patients with alcoholic KS commonly produce intrusions (23, 33), which are similarly to our patients semantic (23). In previous studies of neuropsychologically documented non-alcoholic KS, intrusions have been reported in five cases, including the three in our recent report (15, 20, 50), whereas they were absent in one case (51). Intrusions have been shown in other somatic diseases, including thalamic lesions (52), ruptured aneurysm of the anterior communicating artery (53), Alzheimer's disease (54, 55), traumatic brain injury (56, 57), Parkinson's disease (58), frontal lobe damage (58, 59), and non-alcoholic liver disease (60). In psychiatric diseases, intrusions have been shown in schizophrenia (61), methamphetamine dependence (62), and in attention-deficit hyperactivity disorder (63). Regarding the psychiatric diagnoses of our patients, intrusions are uncommon in bipolar disorder (49), and they occur in major depression only together with anosognosia for the memory impairment (64).

Thus, the presence of intrusions suggests that the memory impairment of our patients has an organic etiology. The lack of evidence for other organic disorders and the semantic nature of the intrusions support the KS diagnosis. Our results suggest that assessment of intrusions in neuropsychological testing might be useful in the diagnosis of non-alcoholic KS. Since intrusions occur very rarely in neuropsychological testing of healthy persons, their presence could be particularly useful when the KS memory impairment is not pronounced or isolated. However, further studies are needed before testing for intrusions can be confidently implemented in the clinical diagnosis of KS. The occurrence of intrusions in both alcoholic and non-alcoholic KS supports the notion of their similarity (6). However, the possible preferential impairment of executive functions in alcoholic KS suggests that important differences may exist between alcoholic and non-alcoholic KS (16). Studies with a larger number of patients are needed to further explore these issues.

Immediate and delayed recall were impaired in most previous studies of neuropsychologically examined non-alcoholic KS (15, 16). Similarly, all patients in the present report had impaired delayed recall, and two patients had impaired immediate recall. However, comparing the results of delayed and immediate memory tests may not differentiate impairment of memory consolidation from impairment of memory encoding (65, 66).

There are only three previous DTI studies in KS. In alcoholic KS, reduced FA-values have been reported in the corpus callosum and the anterior corona radiata in one study (29) and in the fornix in another study (34). We have recently reported on two psychiatric patients with non-alcoholic KS and reduced FA-values in the UF, the corona radiata, and the inferior longitudinal fasciculus (15). Two of our present patients had clear damage in the UF, as assessed by DTI. The third patient had DTI findings that were suggestive of damage in the UF. One patient also had damage in the subgenual cingulum, and in the genu and in the body of the corpus callosum. According to a recent meta-analysis of DTI in affective disorders, decreased FA-values are found only in the genu of the corpus callosum and in the left posterior cingulum (67). Thus, it is unlikely that our DTI findings are due to psychiatric disease. It has been suggested (26), albeit not consistently (68), that the subgenual cingulum contains fibers connecting the anterior thalamus with the posterior cingulate cortex, and the anterior thalamus is commonly damaged in KS (69). Damage in the subgenual cingulum can cause memory impairment (70). Further, reduced integrity of the subgenual cingulum may cause dysfunctional emotional reactivity (71) and in trauma-exposed women hypervigilance (72). Interestingly, dysfunctional emotional reactivity that emerged after the WE has been relevant in the psychiatric treatment of our patient with subgenual cingulum damage.

The most consistent DTI finding in the present report was damage in the UF. The UF is anatomically related to the limbic system, a primary site of neural damage in WKS (73), and damage in limbic regions can cause UF abnormalities (74). This is consistent with the notion that the UF abnormalities in our patients were caused by prior thiamine deficiency. The UF is involved both in emotion regulation and in memory function (74, 75). Therefore, the UF could be particularly relevant in memory impairment of psychiatric patients. Most importantly, UF damage is preferentially associated with impairment in verbal immediate and delayed recall (74). These types of memory were affected in our patients, similarly to patients with alcoholic KS (76). In Patient 1, verbal working memory was also impaired; interestingly, in a study of patients with schizophrenia (which is associated with reduced UF FA-values), UF damage was related to worse performance in working memory tasks (77).

Neuropsychological and imaging studies have suggested that intrusions are associated with frontal and temporal lobe dysfunction (59, 78, 79); our findings of intrusions and damage in a major frontotemporal tract (UF) support that notion and extend it to non-alcoholic KS in the context of impaired brain connectivity. Together with results from our previous study (15), our present findings suggest that structural white matter abnormalities, particularly in the UF, may be a feature of non-alcoholic KS in psychiatric patients.

Two patients have OSA that was treated at the time of the neuropsychological examination. Untreated OSA can affect immediate and delayed verbal recall, but there is no effect on recognition memory (80). Patients with treated OSA can still have cognitive impairment, but it is mostly restricted to executive functions (81). It is possible that the cytostatic drugs used in the leukemia treatment have contributed to the cognitive impairment of Patient 1 (82, 83). Deficiency of other micronutrients may have aggravated the effects of thiamine deficiency (84).

Patient 3 has had prominent gastrointestinal symptoms since the WE episode. Gastrointestinal manifestations of thiamine deficiency (“gastrointestinal beriberi”) have been reported (85, 86). Short-term experimental thiamine deficiency in humans caused transient gastrointestinal symptoms and radiologically documented reduction in gastrointestinal motility (87). To our knowledge, there are no previous studies showing chronic non-dysphagia gastrointestinal symptoms or motility impairment after thiamine deficiency. Thus, it is difficult to be certain that the chronic gastrointestinal symptoms in our patient are a manifestation of WKS.

Patient 1 developed muscle weakness during WE, which has persisted for nine years. Muscle weakness occurs in non-alcoholic WE (88), and it can be a prominent manifestation (89). Residual muscle weakness has been reported after non-alcoholic WE (90, 91).

Limitations of our study include the small number of patients and not testing retrograde memory nor for spontaneous confabulations. In addition, the conventional MRI examinations were evaluated clinically, and no quantitative analysis was performed. Results of the DTI-analysis may be influenced by the fiber tracking methodology used (92). There is strong evidence that in non-alcoholic WE brain involvement is considerably more widespread compared to alcoholic WE (93). Therefore, in our study of non-alcoholic KS we examined a large number of individual tracts. In addition, we divided two large tracts (corpus callosum and cingulum) into their components, and based on pathology findings (11) we examined separately the whole fornix and its descending part. This approach enabled us to find damage in the UF, and in parts of the corpus callosum and the cingulum. However, studying a large number of tracts in a small number of patients increases the probability of chance findings. The tracts that were found damaged are important in memory function and damage in the UF was found also in our previous study of non-alcoholic KS (15), both of which increase confidence in the biological relevance of our findings. Nevertheless, our DTI analysis should be considered exploratory. Moreover, we did not assess frontocerebellar tracts and the mammillothalamic tract, or comprehensively assess thalamocortical connections. Larger studies are needed to better understand the role of brain connectivity in KS and to further assess the usefulness of DTI in its diagnosis. A strength of our study is the long follow-up, which provides important insight into the natural history of non-alcoholic KS.

In conclusion, our results expand the diagnostic spectrum of non-alcoholic KS to psychiatric patients with variable neuropsychological findings. Our results are in accord with previous findings of a variable clinical presentation of KS, particularly broad cognitive impairment. Our observations suggest a need to comprehensive evaluate psychiatric patients with a history of probable WE for subsequent KS, and in particular to consider the possibility of KS in patients with severe but not debilitating effects of memory impairment on everyday functioning. Our findings also suggest that frontotemporal connectivity may be impaired in non-alcoholic KS in psychiatric patients. Combining with results of our previous study (15), six psychiatric patients with non-alcoholic KS have been identified from a single physician's practice during a period of a few years. This, together with the dearth of previously reported cases and the established notion of KS underdiagnosis, suggests that large-scale screening of psychiatric patients for WKS could result in the identification of many more cases. This, in turn, could contribute to more appropriate treatment of these patients and offer possibilities for significant advancements in memory research.

## Author contributions

GN conceived of the study, reviewed the literature, and is responsible for overall study design. TI designed and assessed the neuropsychological examinations. TK designed and assessed the MRI/DTI examinations. JP performed the neuropsychological examinations. SP reviewed results of neuropsychological examinations. GN, TK, SP, RV, and TI interpreted results. GN drafted the manuscript, which was critically revised by all authors. All authors read and approved the final version of the manuscript.

### Conflict of interest statement

The authors declare that the research was conducted in the absence of any commercial or financial relationships that could be construed as a potential conflict of interest.
